# Electroactive polymers for sensing

**DOI:** 10.1098/rsfs.2016.0026

**Published:** 2016-08-06

**Authors:** Tiesheng Wang, Meisam Farajollahi, Yeon Sik Choi, I-Ting Lin, Jean E. Marshall, Noel M. Thompson, Sohini Kar-Narayan, John D. W. Madden, Stoyan K. Smoukov

**Affiliations:** 1 Department of Materials Science and Metallurgy, University of Cambridge, Cambridge CB3 0FS, UK; 2 EPSRC Centre for Doctoral Training in Sensor Technologies and Applications, University of Cambridge, Cambridge CB2 3RA, UK; 3 Advanced Materials and Process Engineering Laboratory, University of British Columbia, Vancouver, British Columbia, Canada V6T 1Z4

**Keywords:** sensor, electroactive polymer, conducting polymer, dielectric elastomer, liquid-crystal elastomer, piezoelectric polymer

## Abstract

Electromechanical coupling in electroactive polymers (EAPs) has been widely applied for actuation and is also being increasingly investigated for sensing chemical and mechanical stimuli. EAPs are a unique class of materials, with low-moduli high-strain capabilities and the ability to conform to surfaces of different shapes. These features make them attractive for applications such as wearable sensors and interfacing with soft tissues. Here, we review the major types of EAPs and their sensing mechanisms. These are divided into two classes depending on the main type of charge carrier: *ionic EAPs* (such as conducting polymers and ionic polymer–metal composites) and *electronic EAPs* (such as dielectric elastomers, liquid-crystal polymers and piezoelectric polymers). This review is intended to serve as an introduction to the mechanisms of these materials and as a first step in material selection for both researchers and designers of flexible/bendable devices, biocompatible sensors or even robotic tactile sensing units.

## 1. Introduction

Electroactive polymers (EAPs) are polymers that undergo shape and/or dimensional change in response to an applied electrical field [1,2]. EAPs are a subset of electroresponsive polymers (ERPs), which exhibit electrically coupled responses in general [3]. The earliest study on EAPs was performed by Roentgen [4] and Sacerdote [5], who were working on the deformation of a dielectric polymer induced by an electric field. EAPs are attractive to people working on electrically driven soft actuators [1,2], as some EAPs such as dielectric elastomers [6,7] can handle much larger strains than their equivalent conventional actuator materials, such as piezoelectric ceramics. EAP artificial muscles are strongly comparable to biological muscle due to their response to electrical stimulation, though their operation mechanism is significantly different [1,8]. Besides actuators, EAPs have revealed their potential in other applications [1,2,8–11] such as sensors, electronic components and energy generators. EAPs are suitable for sensory applications ranging from haptic sensing [10] to blood pressure and pulse rate monitoring [12] and even chemical sensing [13]. This is due to numerous favourable properties: facile fabrication, high mechanical flexibility, customizable electromechanical coupling properties and tailorable geometries [1,2,8–11]. Furthermore, coupling EAPs into micro-electro-mechanical systems (MEMS) has been discussed by both the EAP [14] and MEMS [15] communities.

EAPs can generally be classified into two categories: ionic EAPs and electronic EAPs [1]. The electrical activation of ionic EAPs [1] is due to the migration of ions or molecules (solvents). Some examples of ionic EAPs are conducting polymers (CPs) [16,17] and ionic polymer–metal composites (IPMCs) [18,19]. Electronic EAPs [1] are activated by applied electric fields and Coulomb forces. Dielectric elastomers [20,21], electrostrictive polymers [22], liquid-crystal polymers [23,24] and piezoelectric polymers [25] belong the electronic EAP category.

EAPs comprise a family of promising materials for sensing, but are still new to most researchers working on sensor-related topics. Since there is no material that can cover all sensory requirements, the selection of a material should be made carefully to suit individual requirements [2]. EAPs should certainly be considered complementary to conventional sensing materials, especially in areas where high strains and conformity to soft materials is desired. In this article, we review such materials and aspects of their associated sensing mechanisms. Major categories of ionic and electronic EAPs in sensory technologies are discussed, along with a number of illustrative examples.

## 2. Ionic electroactive polymers for sensing

### 2.1. Introduction to ionic electroactive polymers

Ionic EAPs usually work at low voltages (less than 5 V [11] for actuation). Additionally, many materials used in the synthesis of ionic EAPs, such as polypyrrole (PPy) and poly(3,4-ethylenedioxythiophene) (PEDOT) are usually biocompatible. Thus, such EAPs are suitable for applications in biological environments [2]. Ionic EAPs normally require an ion reservoir to operate, so that ions or molecules can be transported within them. Owing to the presence of ions in these EAPs, they share some common features as responsive materials: (i) the stress and/or strain applied to these materials will cause ion migration and perturb the charge distribution; (ii) the properties of ionic transport into electrodes and/or through an electronic separator will influence the overall outcomes in applications such as sensing and actuation. Further discussion on their advantages and disadvantages will be given in table 3. Most ionic EAPs can be further categorized into CPs, IPMCs and polymer gels [1,2]. Carbon nanotubes [26] have recently been added to this group as well [27]. Here, we focus on CPs, IPMCs, carbon nanotubes and their derivatives.

### 2.2. Sensors based on conducting polymers

CPs or intrinsically conducting polymers (ICP) are organic polymers that are electronically conductive with relatively high and reversible ion storage capacity. The mechanisms of both mechanical sensing and actuation are similar and based on the insertion and expulsion of ions into and from the polymer structure—the structure itself being ionically as well as electronically conductive [28]. Depending on external stimuli and the produced output, this kind of material can be used as an actuator or sensor.

Two different configurations have been used for CP-based sensor devices. One is a free-standing film of CPs, which operates in an electrolyte. Another configuration is the trilayer structure, which is made of two CP layers at the top and bottom, with an electrically non-conductive separator layer between them. The separator layer, which is ionically conductive, works as an ion reservoir and also as an electrical insulator. Trilayer sensors with an electrolyte within a separator layer can function in air and do not need an external electrolyte.

#### 2.2.1. Free-standing films of conducting polymers

**Figure 1.**
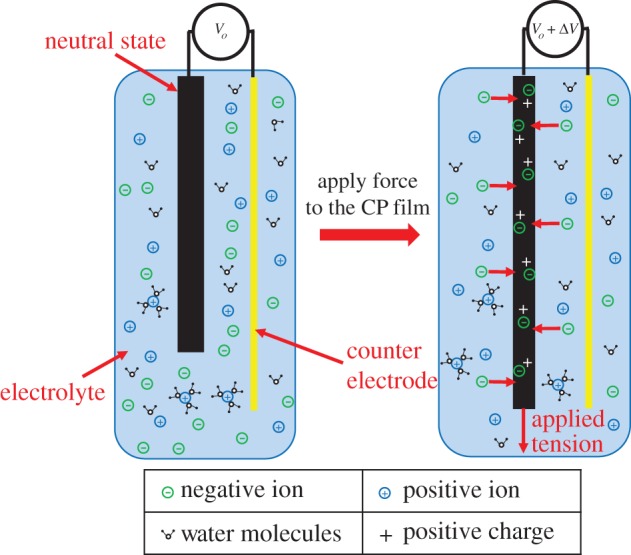
Schematic of free-standing film of conducting polymer as a linear force sensor before and after applying tension.

The mechanochemoelectric effect (which results in ionic and electronic currents produced by mechanical deformation) was observed in a free-standing film (figure 1) of conducting polymer by Takashima and co-workers in 1997 [29]. In this process, mechanically induced charge is converted into electrical energy. This energy is proportional to the magnitude of the applied load and the dimensional change of the film. The efficiency of this conversion was estimated to be less than 0.01%, and the induced voltage was of the order of a few microvolts [29]. It was also shown that this mechanochemoelectric phenomenon is reversible and that the induced charge for all cycles was nearly the same. CPs' ability to measure relatively large strains (10 times larger than typical piezoelectric sensors) and their low mechanical impedance (Young's modulus) makes this kind of sensor potentially useful for multiple applications such as instrumentation to detect strain and force [30]. Additionally, tensile strain can be measured by a free-standing film of CP.

There are two hypotheses dealing with the mechanism behind this voltage generation. In one suggested mechanism, if the conducting polymer film or layer is compressed, the mechanical deformation leads to an increase in the concentration of mobile ions relative to the ionic concentration in the external electrolyte. Typically, the mobile ion species is either cationic or anionic, with charge being balanced by the electronic charge on the polymer backbone or by bulky, immobile counter-ions. This concentration difference and the mechanical stress on the mobile ions cause net expulsion of the mobile ions from the polymer structure and generate a voltage difference that is detectable by open circuit measurements [31]. Similarly, increasing mechanical tension in the polymer structure increases the volume of the polymer, leading to a decrease in ion concentration. This causes an influx of mobile ions of one type into the polymer (anions are shown in figure 1), leading to a voltage difference. An alternative theory suggests that ions are inserted or expelled directly as a result of mechanical stress, not due to changes in concentration [30]. This applied mechanical stress can be related to generated voltage by CP sensors as an output.

A linear relationship between generated voltage and applied stress was reported by Shoa *et al.* [30] for a PPy free-standing film. The generated voltage Δ*V* is related to applied stress, *σ* by the following equation: Δ*V* = *α_s_σ;* where the strain to charge ratio for sensing, *α_s_* is an empirical linear coefficient. The sensor response is relatively stable in the frequency range 0.1–100 Hz.

Based on the sensing mechanism in CPs, counter-ions play an important role in the conversion of mechanical energy to electrical energy, and vice versa. Madden [32] also predicted the charge generation due to mechanical deformation in CPs. He also mentioned the relationship between voltage polarity (±*V*) and the type of counter-ion [32].

Ergonomic comfort, elasticity, high thermoresistivity and piezoresistivity of the PPy conducting polymer have attracted some researchers to use it in wearable e-textiles in biomedicine [9] and wearable electronics [33]. Examples include the deposition of a thin layer of PPy on a Lycra/cotton fabric to make a sensitive glove [34], and coating elastomeric fabric to make an intelligent knee sleeve to provide feedback on knee flexion angle [35]. Although the material has several interesting properties in the context of wearable devices, its long response time to reach steady state (in a few minutes) is the major limitation for practical usage [9].

Gas or chemical sensors are another interesting application for CPs, as the absorption of gas molecules leads to a change of electrical conductivity in the polymer matrix. Compared to sensors based on metal oxides, conducting polymer-based chemiresistors have several improved characteristics such as higher sensitivity and shorter response times [36]. The sensitivity of the PPy conducting polymer to ammonia, nitrogen dioxide, carbon dioxide and organic vapours, such as alcohols and ethers has been reported [36–39].

CPs have also been employed in the design and fabrication of biosensors [40]. Facile electro-polymerization, and their potential for miniaturization and functionalization by doping or grafting, make CPs good candidates for use as suitable substrates for DNA sensors [41,42]. Meanwhile, stability, ability to be modified by enzyme to exhibit different analytical characteristics, and electrode protection from fouling and interfering material are some advantages of CPs for electrochemical biosensing applications [43].

Conducting polymer free-standing films can be produced by electrodeposition, as is often done with PPy [44]. Low-temperature deposition produces films with good electrical and mechanical properties [30,44]; these are doped, during growth, with anions. Although anions often constitute the mobile charges, large anions such as dodecylbenzenesulfonate (that are essentially immobile) can be inserted during electrodeposition [45–47], creating cation-transporting sensors. The detected voltage in cation-transporting sensors is in opposite polarity when compared with anion-transporting sensors. Unlike PPy, polyaniline is soluble, and can be cast to create films [29]. In chemical deposition, PEDOT, PPy and polyaniline can also be deposited from the monomer in vapour or liquid phase, in the presence of an oxidizing agent that drives polymerization on or within a substrate [48–50].

#### 2.2.2. Trilayer structure based on conducting polymers

**Figure 2.**
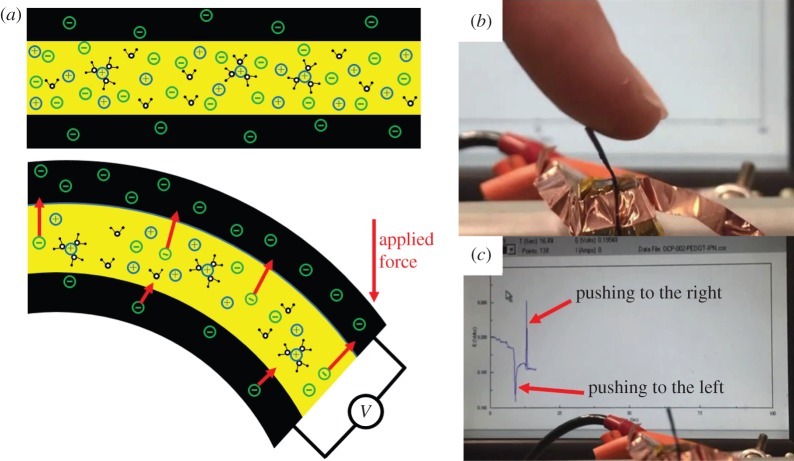
Schematic of (*a*) a bending trilayer sensor. In (*b*), a trilayer sensor is deflected by a finger, and (*c*) shows an example of measured open circuit potential generated by brief mechanical stimuli. The direction of the peak (up or down) is related to the direction of mechanical excitation. The magnitude of voltage spikes in this example is approximately 10 mV. Pushing a trilayer to the left generates an upward peak, while pulling the trilayer to the right generates a downward peak, with the positive electrical connection on the (left/right) side. A video about the tri-layer conducting polymer sensor and actuator is available in the electronic supplementary material.

Figure 2 shows a bending trilayer sensor, with the expanded layer being penetrated by mobile ions, while mobile ions are expelled from the contracted layer on the opposing side due to mechanical stress and the increase in ion concentration. Stress effects may also occur in ions in the separator layer. The generated potential difference between two layers can be detected by an open circuit potential measurement, or by detecting a short circuit current. The voltage difference produced is given by the strain to charge ratio multiplied by the applied stress. Typical values of strain to charge ratio are 10^−11^–10^−10^ m^3^ C^−1^, so that a 1 MPa stress produces voltages between 0.02 and 0.06 mV, and large charges of between 2000 and 6000 C m^−3^ [30,32]. The charge produced in thin films is large compared with piezopolymers (piezoelectric polymers, which convert pressure to voltage—refer to §3.4).

A mechanical bending-type trilayer sensor using PPy as the CP layers was investigated by Wu *et al.* [31]. They observed millivolt signals in response to millimetre deflection, which generated 1000 C m^–3^ charge for 1% strain in the PPy film. Sinusoidal voltage output was detected in response to sinusoidal displacement excitation. A linear relationship between induced strain and charge density during bending deformation of the trilayer sensor was observed. They also reported that the strain to charge ratio is the ion-dependent parameter and is different for different ions, and sensor response can be improved by using ions that have larger strain to charge ratio.

Tensile and compressional strain can also be measured by conducting polymer-based sensors in trilayer configurations. PEDOT conducting polymer was used as a mechanical sensor in a trilayer configuration [51]. The output voltage in response to ±2% sinusoidal strain was measured at approximately 0.20 mV, with the same frequency as the input signal. A linear relationship between applied strain and the output voltage (in open circuit potential measurement) was reported.

Interesting results by Otero and his team [52] report the change in consumed electric energy by a trilayer PPy-based device in response to changes in load. It was observed that during the application of a constant current between two PPy electrodes in a trilayer arrangement, there is a linear increase in consumed electrical energy with respect to load. They also reported that this energy drops linearly with respect to increasing temperature. These properties allow this device to be used as a sensor for both mechanical load and temperature. In a similar study, the tactile sensitivity of the trilayer was investigated. The PPy trilayer was used to push an obstacle, and a linear relationship between applied potential and obstacle weight was obtained [53]. The materials' key properties have been extensively characterized by different groups. These properties include sensitivity (the minimum detectible physical signal) [54,55], linearity (linear relationship between input and output) [56], their operation at the micro-scale [55,57] and frequency response [58].

**Figure 3.**
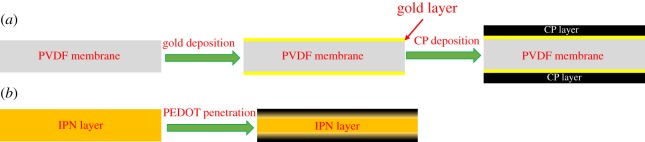
Schematic of fabrication of conducting polymer-based trilayers (*a*) CP/Au/PVDF/Au/CP structure, gold is sputtered on the PVDF membrane and then the CP layer is deposited by electrochemical deposition. (*b*) PEDOT/IPN/PEDOT structure, PEDOT is polymerized inside the IPN film by chemical deposition, which creates a PEDOT penetrated layer at the top and bottom of the IPN film with density gradient of PEDOT towards the surface.

Trilayer structures (figure 3) are formed with porous or ionically conducting material as the substrate, onto which two layers of conducting polymer are applied (top and bottom).

Wu *et al.* [31] and subsequent papers from Alici and colleagues [56,58,59] employ porous polyvinylidenedifluoride (PVDF) as the substrate, onto which a thin layer of platinum (or gold [59]) is sputtered on each side. PPy films are electrochemically deposited on the metal layers. Microstructures can be produced by creating thin porous layers of PVDF [31,60,61]. An approach that was used by a team at the University of Cergy-Pontoise involved the synthesis of films containing ionically conductive poly(ethylene oxide) (PEO) interpenetrated with an elastomer (nitrile butadiene rubber). The latter affords mechanical stability, imparting high mechanical elasticity and ionic conductivity to the resulting interpenetrating polymer network (IPN) [48,51,62]. The EDOT monomer is deposited onto the substrate surface. Following penetration into the substrate, it is polymerized to create a very robust and ionically conductive trilayer sensor/actuator combination [48,63–65]. Recent work has shown a resonant frequency of 900 Hz in an actuated microstructure [62]. Freedom from delamination is one of the major advantages of PEDOT/IPN/PEDOT trilayer structures for long-term operations (more than 3.5 × 10^6^ cycles).

Trilayers based on CPs deposited on PVDF or PEDOT/IPN/PEDOT trilayers can be swollen with ionic liquids or other salts dissolved in different electrolytes to function in air as a sensor (e.g. 0.1 M LiTFSI in propylene carbonate [66–68]). Stiffness, deflection, current and frequency response are tailored by varying length, width and thickness.

Otero and Cortés fabricated a very simple triple layer sensor; they electrodeposited the PPy film on AISI 304 stainless steel. To control the adherence to the metal, the morphology and the stability, they applied square wave potentials between –0.3 V (2 s) and 0.872 V (8 s). After deposition they taped PPy-deposited film on stainless steel with double-sided plastic tape, and peeled off the film and taped the other side of the tape to new PPy-deposited film and peeled it off again. Finally, they made the PPy/double-sided plastic tape/PPy triple layer sensor [52,53].

### 2.3. Sensors based on ionic polymer–metal composites

**Figure 4.**
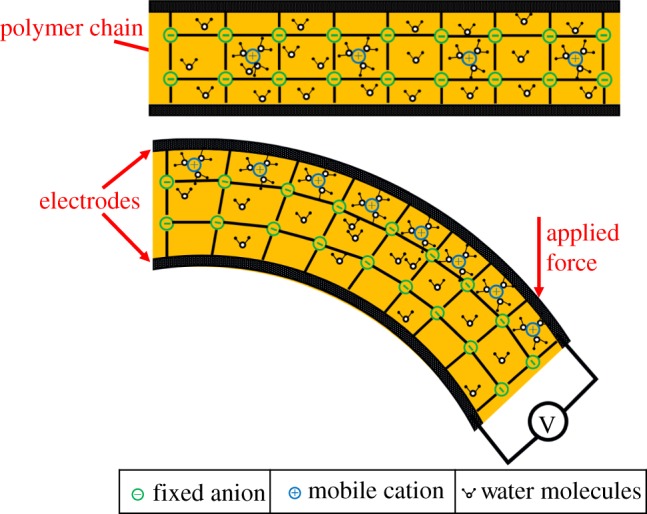
Schematic of an IPMC sensor. Application of force leads to bending of the IPMC and expansion (dilution of ions) at the top and contraction (concentrating ions) at the bottom, which causes a concentration gradient. Difference in concentration leads to migration of mobile cations (surrounded by water molecules) towards the diluted section. Owing to change of charge amount carried by cations between electrodes, potential difference is generated between the electrodes. This potential can be measured by open circuit voltage.

Similar to the CPs, IPMC sensors and actuators also work based on the movement of ions, but with different mechanisms and structures. IPMCs have trilayer structures, involving a combination of ionic polymers and metallic electrodes. IPMCs are reported as both sensors and actuators [10,19,69–71]. Working as sensors, their great sensitivity to physical stimuli such as mechanical force (which can induce bending on the IPMC sensors, figure 4) makes them very attractive [72,73]. Unlike sensors based on CPs, IPMCs are mainly used as mechanical sensors. Therefore, this section mainly focuses on mechanical sensing based on IPMCs.

In 1970s, Grodzinsky & Melcher [74] demonstrated electromechanical transduction in collagen membranes. They proved experimentally that both mechanical-to-electrical and electrical-to-mechanical transductions are possible in the polyelectrolyte membranes used [74]. IPMC-based vibration sensors were reported by Sadeghipour *et al.* in 1992 [75]. The output voltage versus applied tip displacement upon bending a cantilevered IPMC was measured by Mojarrad and Shahinpoor [76]. The magnitude and direction of the output voltage were found to be dependent on the magnitude and direction of the forced-induced deformation. Another experiment using IPMC as a mechanical sensor was conducted by Ferrara *et al.* [77].

IPMC is normally established in a trilayer fashion with the metallic electrode attached on the top and bottom of an ionic polymer layer [78,79]. Ionic polymers are considered polyelectrolytes, formed by a fixed, covalently bound network of immobile ionic repeat charge units (hydrophilic). These are covalently bound to repeat units which are sometimes grafted onto a network of a non-ionic polymer (hydrophobic). The hydrophilic, microstructured network creates porosity, enhancing the charge transport of oppositely charged mobile counter-ions when swollen in the presence of diluent [69] (figure 4). Acidity, or the ion exchange capacity (IEC), of the ionic polymer membrane indicates the capacity for the counter-ions storage; the ion conductivity of the ionic polymer indicates ion mobility across the membrane [69]. IEC and ionic conductivity are the two major characteristics of ionic polymers as sensors and actuators. Both of them depend on the structure of the membrane. Meanwhile, the ionic conductivity is also related to the size and charge of the counter-ions as well as electrolyte type and uptake [80]. However, the tensile modulus usually decreases when ionic conductivity is increased by higher diluent uptake. The main research efforts in this area are to synthesize ionic polymers with higher ionic conductivity (operating in both hydrated and dry conditions), improved stability (chemical and thermal) and enhanced mechanical properties (e.g. strength) [69,81]. DuPont's Nafion [82], Flemion, Aciplex, Aquivion (Hyflon) and other synthesized sulfonated aromatic ionic polymers have been reported as polyelectrolytes for IPMC sensing and/or actuation [69]. A typical ionic polymer consists of perfluorinated alkenes with short side chains terminated by ionic groups (e.g. sulfonic or carboxylic acid groups for cation exchange, or ammonium cations for anion exchange). The volume proportion of polymer backbones determines the mechanical strength of IPMCs: the higher the volume proportion, the higher the mechanical strength. Metallic electrodes can be formed by either chemical reduction (such as electroless plating [83]) or physical deposition (such as sputtering [84]). Metals that have been used successfully as electrodes are platinum [85,86], copper [87], silver [83], palladium [84] and gold [88,89].

The theory behind IPMC mechanical sensing was covered in a handful of review papers [18,69,78,90]. Two methods to measure mechanical deformation have been proposed, as described below.

In the first method, the generated potential difference between electrodes is measured. The IPMC's mechanical sensing properties were explained by the charge imbalance of ion migration by mechanical deformation [19]. When the external force is imposed on the IPMC sensor, due to the stress/strain gradient, shifting of mobile cations becomes possible and they move towards the expanded region to balance the concentration of ions. The gradient of charge along the thickness of the IPMC sensor (figure 4) generates a potential difference which can be detected by a low-power amplifier or open circuit potential measurement [18,91,92]. The hypothesis behind this mechanism of sensing is that the charge density is proportional to the induced strain [93].

In the second measurement method, the surface resistance of the metallic electrode of IPMC is measured. This resistance changes with expansion and contraction of the electrodes. When the electrode is stretched the resistance increases, and compression of the electrode decreases the resistance. In the case of bending IPMCs under applied force or strain, the resistance of one side increases while the other side decreases. This difference between resistances of both surfaces is correlated to the bending curvature and also increases cumulatively. The measured resistance difference between electrodes is used to calculate the radius of curvature, which leads us to find the applied strain. A four-probe system is employed to measure the surface resistance of the electrodes [94,95].

IPMCs can be used for both static and dynamic mechanical sensing. They demonstrate potential in sensing curvature variation for engineering structures [96], fluidic flows [97], force [98] and even the inclination (angle change) of a body [99]. Though the electromechanical coupling properties of IPMCs are found to be influenced by the solvent [100,101] and other conditions such as the humidity of the environment [102], chemical sensing based on IPMCs is rarely explored. Some IPMCs are soft and non-toxic hydrophilic materials, which show great potential for biomedical applications. Multifunctional tactile sensors [103], muscle movement detectors [104], blood pressure, pulse rate and rhythm sensors [12], pressure sensors in human spines [77] and hand prosthetic applications [10] based on IPMCs have been demonstrated.

### 2.4. Sensors based on carbon nanotubes

Application of voltage to carbon nanotube electrodes immersed in an electrolyte results in charge to the electrodes. This charge is balanced by the counter-ions from the electrolyte. Insertion/expulsion of ions into/from the carbon nanotubes can generate positive and negative strain and enable carbon nanotube electrodes to work as an actuator. Inversely, doping carbon nanotubes with some molecules can produce a potential difference or change the electrical conductivity. Change in nanotube structures or change in electrical conductivity can be sensed and enable carbon nanotubes to be employed as sensors.

Carbon nanotubes (CNTs) have drawn much attention due to their unique properties, including their one-dimensional nature, high stiffness and strength, thermal conductivity, ballistic transport and high surface area [105]. CNTs exhibit actuation and sensing behaviour due to their ability to store both charge and molecules. Storing charge in carbon nanotubes causes change in their length and produces strains of up to 1%. Conversely, charged CNTs in the form of yarns can generate voltage when tensile stress of up to several hundred megapascals is applied [106]. In the case of the force sensor, similar mechanisms to CPs have been suggested for CNT-based sensors. Axial mechanical tension increases the length of the yarns, which leads to radial compaction. This reduction in diameter decreases the available inner surface area for ion interaction and causes expulsion of interior ions. The ion expulsion produces an inward current into the yarns explained by Mirfakhrai *et al.* [106]. Chemical vapour deposition (CVD), arc-discharge technique and laser ablation are the main methods used in the preparation of the CNT layer [105].

**Figure 5.**
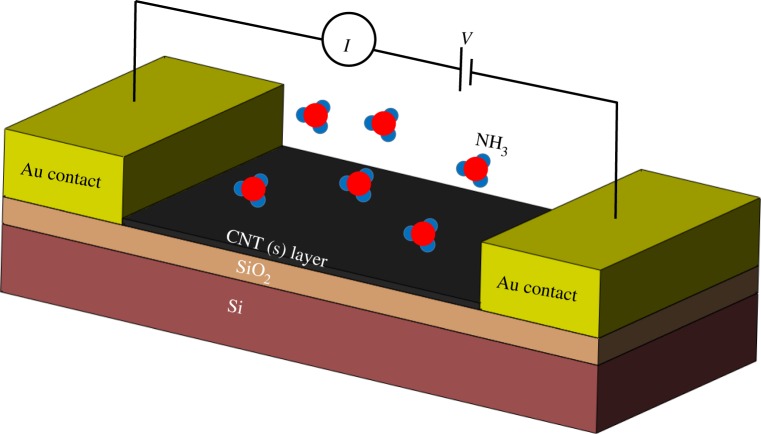
Schematic of a CNT gas sensor. The CNT layer is used between two electrodes, and the current response to constant applied voltage is measured. Change in resistance of the CNT layer, which causes change in current, shows gas absorption.

Very large surface-to-volume ratio means the carbon nanotubes have high adsorptive capability: ideal for use in gas or chemical sensors. As the electronic properties of the nanotubes change with atomic structure and chemical doping, they are suitable for sensor miniaturization while maintaining high sensitivity. As shown in figure 5, when the gas molecules are absorbed by the CNT(s) layer and work as dopants, the conductivity of this layer changes, which causes variation in resistance between two Au contacts [105,107,108].

CNTs have been employed in electrochemical sensing to enhance sensitivity, especially in biosensing applications [109,110]. Excellent conductivity, absorptivity and biocompatibility are other advantages of CNTs as electrochemical biosensors [110–114].

### 2.5. Sensors based on other ionic electroactive polymers

Other ionic EAPs, such as hydrogels [2,115,116], have also been studied for sensing applications. The sensitivity of the hydrogels to some physical parameters such as temperature [117], pH [117,118], electrical voltage [119], salt concentration [120] and concentration of organic materials in water [121] make them interesting materials for using in chemical sensor applications and biosensors such as DNA and protein detection [122].

Smart hydrogel or stimuli-responsive hydrogels can change their volume by more than one order of magnitude and reversibly convert chemical energy into mechanical energy [119]. Using hydrogel with different techniques for sensor applications has been reported [40,123]. Swelling the hydrogel, which causes bending of the thin silicon plate to change the output voltage, is one of the demonstrated approaches. Adding a hydrogel layer to the micro-pressure sensor can help to monitor the analyte-dependent swelling of the hydrogel to detect the change in solution. The diffusion-driven mechanism of the hydrogels gives them slow response times, but this can be improved through miniaturization. The response time of such materials is affected by the viscoelastic and hysteresis behaviour of the hydrogel [123].

## 3. Electronic electroactive polymers for sensing

### 3.1. Introduction to electronic electroactive polymers

Unlike ionic EAPs, electronic EAPs are free from any electrolyte medium. No ion migration is required for the electromechanical coupling. Therefore, electronic EAPs require a shorter time period for response or relaxation than ionic EAPs: this can be less than 1 ms [11]. Thus, electronic EAPs can also work at higher frequencies than ionic EAPs for dynamic sensing. Dielectric elastomers, electrostrictive polymers, liquid-crystal elastomers (LCEs, typical flexoelectric polymers) and piezoelectric polymers are the main subgroups within electronic EAPs [1,2]. In the following subsections, we will focus on dielectric elastomers, LCEs and piezoelectric polymers as they are more studied in sensory applications.

### 3.2. Sensors based on dielectric elastomers

A dielectric elastomer (DE) sensor is a type of strain sensor that detects the change of capacitance in a capacitor-like system. In this section, the design and mechanism of DE sensors is briefly introduced, and the benefits and drawbacks are also discussed to help both theoretical understanding and potential application.

DE sensors are based on a system with two compliant electrodes and an intermediate dielectric layer (formed from a long chain polymer with suitable electric permittivity and film thickness). These systems are DE transducers [21,124,125], which include DE actuators [6,126–129], DE sensors [130] and DE generators [131–133].

**Figure 6.**
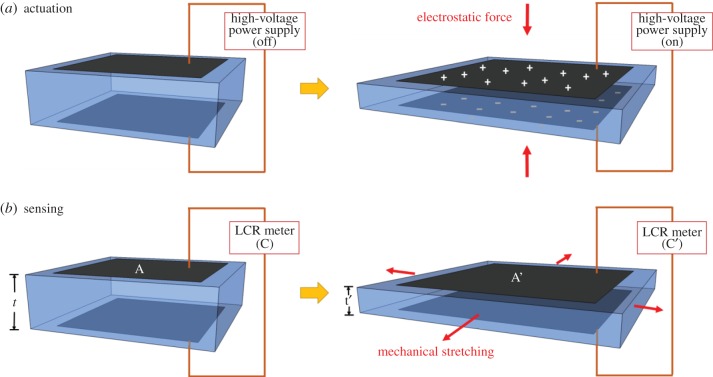
Schematic diagrams show the mechanisms of operation for a DE in (*a*) actuator and (*b*) sensor modes. When applying voltage across the actuator, the attracting force between electrodes makes the DE film contract in thickness but expand in lateral directions. When laterally stretching the sensor, the change in capacitance (C to C’) can be correlated to the strain due to stretching.

Development of EAPs based on DE materials can be traced back to Roentgen's work in 1880 [4]. In his experiment, he observed the deformation of a piece of natural rubber when a high electrical field was applied to it. This is the first reported instance of DE actuation. Figure 6*a* shows the basic concept of DEAs. When a voltage is applied to the two compliant electrodes (black sheets shown in figure 6) at the top and bottom of the elastomer, these two electrodes mutually attract and tend to move towards each other, thus squeezing the elastomer by the electrostatic force (also known as Maxwell stress) and inducing a reduction in thickness. Assuming the elastomer is incompressible with a positive Poisson's ratio, there must be a lateral expansion in the plane normal to the direction of the electrical field. Since the electrodes are compliant, they will expand with the elastomer without electrical disconnection during the deformation process. When the applied voltage is removed, the whole system reverts back to its original state.

DE sensors can have a similar basic design to DE transducers (but without the application of high voltage) to detect changes in strain. This is done by measuring the capacitance across the elastomer after deformation by stretching, relaxing or compressing. Figure 6*b* depicts a typical design of DE sensor. When the system is going through deformation due to applied force, the capacitance created by the electrodes changes with the planar area and the thickness of the system, as described in the following equation: *C* = *ɛ*_0_*ɛ*_r_ *A*/*d*; where *C* is the capacitance, *ɛ*_0_ is the vacuum permittivity, *ɛ*_r_ is the relative permittivity of the DE material, *A* is the overlapping area of the two electrodes on opposite sides of the DE membrane and *d* is the distance between the two electrodes (also the thickness of the DE film). For example, in figure 6*b*, if the system is expanded laterally by applying force in the planar direction (stretching), the capacitance will increase from *C* to *C’* since *A* increases and *d* decreases. Thus, by measuring the change in capacitance with a capacitance meter or an LCR (inductance (L), capacitance (C) and resistance (R)) meter, the amount of deformation can be calculated [6].

For practical cases, DE sensing may not be as simple as this, since the voltage drop across the electrode, due to resistance and current leakage through the dielectric layer, must be taken into account. Recently, a transmission line model reported by Xu *et al.* [134–136] considered the full impedance of the circuit. This model can better analyse output signals at higher sensing frequency, and can also be used to achieve local detection within the same film [134–136].

In an ideal DE sensor system the electrodes must satisfy two requirements: (i) they must not over-constrict deformation of the DE layer; and (ii) they must stay in conformal contact with the polymer layer during deformation in order to guarantee conductivity. Disconnection of the electrodes during large deformations can cause malfunctions in the system. Meeting these two requirements, the electrode materials can be carbon powder [137,138], silver paint [139], metallic thin film [139], carbon grease [128,140], CPs [139], carbon nanotubes [141], hydrogel with electrolyte [142,143] or graphene [144], and conductive elastomer-based compounds [138,145]. Among these, carbon grease is recommended for fast prototyping and is most widely used since it retains high conductivity during large strain. It is also cost effective and easy to apply to different DE layers [146]. On the other hand, the selection of DE layers also affects the performance of sensors. Since the measured strain is directly related to the change in capacitance, the permittivity of the DE layer should be as large as possible to maximize sensitivity [21]. So far, the most common DE material used in this type of sensor is silicone rubber due to its fast response and low (viscoelastic) hysteresis [125].

Compared with conventional materials used for sensors that are relatively stiff and may fail at low strains [125], DEs can provide a larger range for strain sensing, since strains of 300% have been reported in DE transducers [147], which allow their use in haptic communications or tactile displays [124,148]. Additionally, the fabrication of DE sensors is simple and affordable, and their low weight and stability over many working cycles show potential for applications in the micro-robotics field and biomedical applications such as orthotics and prosthetics [124,149,150]. By integrating DE actuators and DE sensors, a self-sensing actuator can be established. The self-sensing DE actuator has been designed and investigated extensively [151–158]. Furthermore, numerous studies have reported this by measuring capacitance during actuation to monitor the change in strain [158–161]. This design paves the way for micro-scale actuators, since independent sensing devices are not needed. Inevitably, there are some aspects in which DE sensors can be improved, such as inhomogeneity of the materials, the temperature dependence of the elastomer's properties, and conversion between strain and stress in some DE sensors [21,162]. Solving these technical challenges would lead to even wider commercial applications for DE sensors.

Owing to DE sensors' high robustness, reliability, well-costed and well-understood manufacturing process, they are probably the most successful EAP mechanical sensors with regard to their commercialization—with numerous patents [163,164] based on DE sensors and transducers. This is further shown in companies like Parker [165] and StretchSense [166] that have developed sensors based on dielectric elastomers; these can detect stretching, pressure, bending and shear, as well as changes in strain and temperature. StretchSense have integrated these sensors with Bluetooth, an example of well-established pre-existing technology, showing the ready adaptability and ease-of-use of DE sensors. The readouts from the products of both companies can also be transmitted directly to a users' mobile phone.

**Figure 7.**
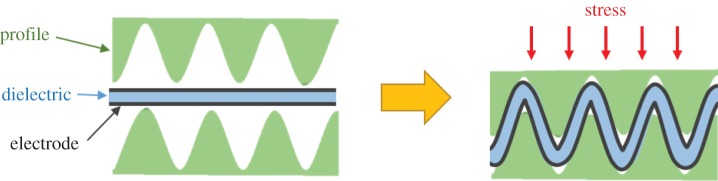
Basic principle of a DE pressure sensor with wave profiles. This figure is based on one previously published in [167].

It has been mentioned by Böse [167] that sensing in the normal direction might be less efficient, compared with sensing lateral stretching. When a compression force acts perpendicular to the film surface, the DE film behaves nearly incompressibly, resulting in a small deformation and a corresponding increase in the capacitance of the flexible capacitor. To better measure the stress load in the normal direction, a novel design has been recently introduced [167]. The basic principle of this sensor is shown in figure 7. The DE elastomer film is embedded between two profiled surfaces in a sandwich configuration. When the sensor mat is compressed, the profiles penetrate into each other, thereby stretching the DE film. This conversion of pressure load to strain leads to a large increase in the capacitance of the DE film, thus enabling detection of stress in the normal direction.

In summary, DE sensors are an additional use of DE transducers. By measuring the capacitance change of a system composed of two compliant electrodes with a DE layer in between, the strain due to an applied force can be measured. It is a good choice due to its high strain and large deformation range measurement, easy fabrication, low-cost, light-weight, repeatability, and also its ability to achieve self-sensing DE actuators in micro-scale applications. Limitations such as inhomogeneity during fabrication also exist. Therefore, proper testing and careful assessment depending on application requirements are necessary when choosing DE sensors as the method to measure strain. Nonetheless, the DE sensor is by far the most successful EAP used so far for mechanical sensing, with well-developed manufacturing techniques and numerous commercial products available in the market.

### 3.3. Sensors based on liquid-crystal polymers

Liquid-crystal (LC) ‘phases' are intermediate phases between the crystalline state (in which molecules are spatially fixed into a very ordered arrangement) and the liquid state (in which molecules are fully disordered) [168]. There are many types of LC phases, and they are categorized by the type and degree of ordering in the molecules making up the material. Increasing disorder can be caused either by increasing temperature, or by adding small solvent molecules. The type of molecules that form LC phases are usually long, rigid rods, and the anisotropy present at molecular level gives LC phases their unusual behaviour. While the LC material is partially disordered, and so retains the ability to flow (like a liquid), it can also display crystal-like optical properties such as birefringence.

If the anisotropic LC molecules are simply chemically joined together to form a long chain, then this ability to flow is hindered and the material simply becomes crystalline; if, however, they are tethered to a long chain molecule which is quite ‘soft’ (i.e. having a low glass transition temperature) then some ability to flow can be maintained. Furthermore, if some chemical linking points are present between these soft chains, then LC phase changes can cause changes in the bulk dimensions of the material. This type of material is termed an LCE [169,170] and can show some really remarkable shape changes; many can shrink by one-third of their length when heated, but shrinking of up to 80% of the original length of the material has been reported [171].

**Figure 8.**
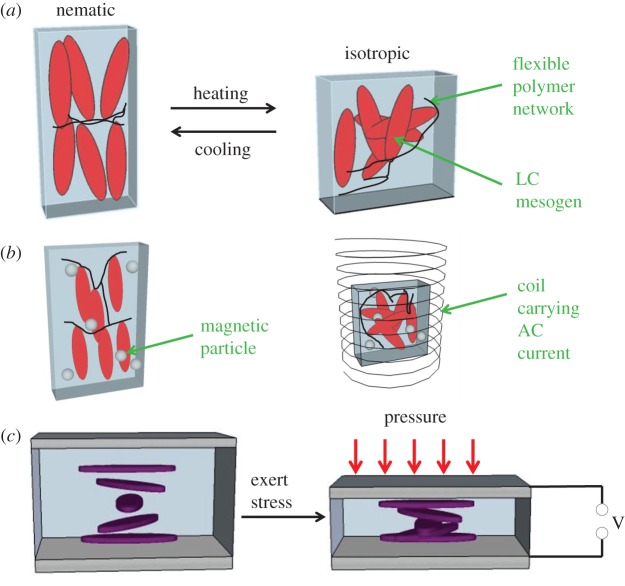
Schematic diagrams to show LCE actuating and sensing behaviour, where (*a*) shows how the ordered ‘nematic’ phase becomes isotropic during thermal-induced actuation, with a corresponding contraction along the LC director; and (*b*) when small magnetic particles are added to the LCE, liquid crystalline order is preserved (particles not drawn to scale). When this material is placed in a rapidly changing electromagnetic field (e.g. inside a solenoid carrying an AC current) the magnetic particles' temperature is raised, causing the same type of contraction as in (*a*). (*c*) A ‘cholesteric’ LCE, with a helical variation in the direction of the LC mesogens, changes pitch when force is exerted in the direction of the helicoidal structure; this can lead to the generation of an electric field and is potentially useful in strain sensing [172]. The mechanism by which this field is generated is discussed more fully in [173].

The schematic in figure 8*a* shows how such a shrinkage can occur; if the mesogens show orientational alignment across the whole of the material (this is termed a ‘monodomain nematic’), then heating causes an increase in disorder and a shrinking of the material along the LC director. This change is reversible. To make LCEs responsive to electric fields, a small amount of a conducting ‘filler’ material (such as carbon nanotubes or magnetic nanoparticles) can simply be added to the LCE, as shown in figure 8*b* [24,174]. In this case, an applied electrical potential causes a flow of current, which heats the material. Then, a dimensional change is caused by the same mechanism as described above. Care must be taken in processing these materials; if the filler is added in too high a concentration, the material can become stiff and dimensional change is hindered.

Certain chiral LCs are, however, sensitive to electrical fields even without an added filler. In order for materials to be ferroelectric (i.e. having a permanent electrical polarization in the absence of an external electrical field), they must fulfil certain symmetry requirements; most types of LC phases have very high symmetry and therefore are not candidates for ferroelectric behaviour [175]. However, in 1975 a team of researchers predicted that in the smectic C LC phase (in which the rigid LC mesogens are arranged in layers, but tilted with respect to the layer direction), and if the molecules are chiral, then a spontaneous polarization arises [175]. This prediction was successfully proven experimentally and has since led to considerable research in the field of ferroelectric liquid crystals (FLCs) [176], which have been commercialized in the field of LC displays due to their rapid switching times. When incorporated into a cross-linked matrix (forming an LCE), they can potentially show piezoelectric effects (i.e. the ability to either produce an electrical current when subjected to mechanical stress, or to show a dimensional change in response to an electrical voltage).

When ferroelectric LCs are chemically bound to a soft polymer network, forming an LCE as described above, the material can undergo contraction when exposed to an external electric field [23,177,178]; the strain changes reported were rather low, approximately 4% in response to applied fields of the order of 1 MVm^−1^ [23]. These materials also tend to be rather soft (with an elastic modulus of the order of MPa), so that, although the applied field causes a strain change, the material is not capable of exerting a large force. Stiffer materials have been fabricated by swelling an LC polymer network to form a gel [179]. These materials have been investigated as electrical actuators (converting electrical signals into a mechanical force) but less extensively for sensing applications. However, some studies on the piezoelectric nature of these materials indicate that surface charges of the order of tens of picocoulombs can be obtained by the application of forces of the order of newtons, and these values are dependent on the temperature at which the procedure is carried out [180].

**Figure 9.**
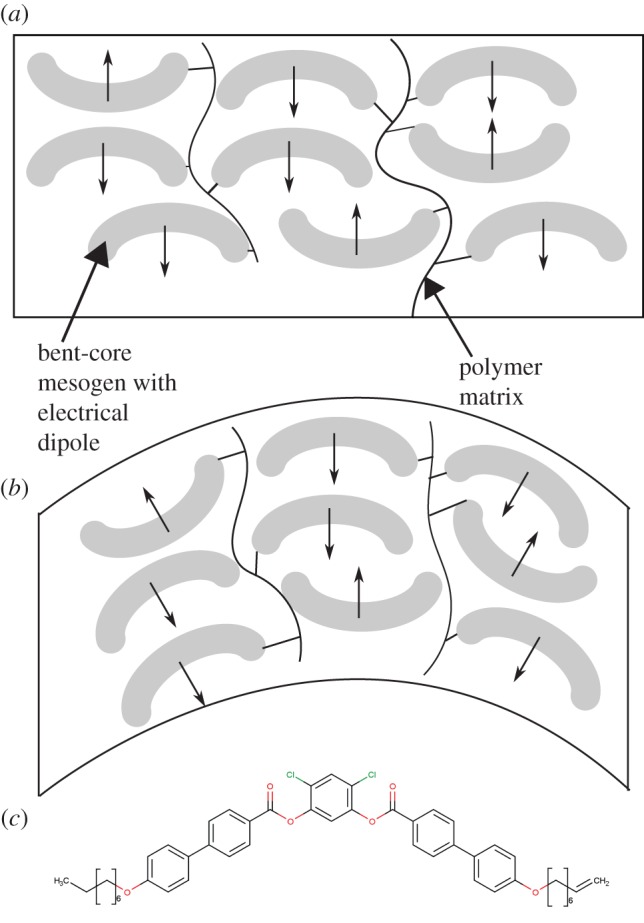
(*a*) Schematic diagram showing a ‘bent-core’ LC mesogen chemically bound to a polymer network. (*b*) A bending distortion creates a change in the overall electrical polarization of the material. (*c*) A typical molecular structure of a polymerizable bent-core mesogen [181–183]. (Online version in colour.)

Strain sensing has also been investigated in the so-called ‘bent-core’ LC polymers. Figure 9 shows schematically how the bending of such a material can lead to an overall change in the electrical polarization, as well as providing an example of a polymerizable bent-core mesogen. As their name suggests, these LCs are rigid mesogenic units that have one bend along their length to form an L-shape; an example is shown in figure 9*c*. This gives rise to novel behaviours, because they can form chiral phases even when the LC mesogens used to create them are achiral. These materials have promise in the field of piezoelectric sensors: under mechanical bending, currents of tens of nanoamperes can be generated [181,182]. This phenomenon has been termed ‘flexoelectricity’ and has been studied both experimentally and theoretically [184].

Piezoelectric effects (which will also be detailed in the next section) have been observed in ‘cholesteric’ LCEs, in which the LC mesogens show helical ordering (and, therefore, chirality). It was predicted theoretically that such cholesteric effects should exist, before experiments showed that piezoelectric voltages of millivolts could be generated [172,173,185]. This type of cholesteric LCE is depicted schematically in figure 8*c*. An interesting recent study showed that cholesteric LC polymers can be made to change their colour in response to humidity and temperature [186].

In summary, LCEs show considerable potential in mechanical sensing due to their tunability and their capacity to undergo large strain changes. Barriers to commercialization include the complexity and expense of their synthesis. While their use as actuators (in which an external trigger causes them to undergo shape change) has been extensively explored, there is considerable scope to extend their use in generating electrical signals in response to external stimuli.

### 3.4. Sensors based on piezoelectric polymers

The term ‘piezoelectric’ originates from the Greek word ‘piezo’, which means ‘pressure’, and refers to the propensity of certain materials to generate electrical charges on their surfaces in response to an applied pressure. Conversely, when an electrical potential is applied across the material, mechanical deformation results.

In general, most of the well-known piezoelectric materials are inorganic [187–190] due to their high piezoelectric strain constant (*d*, the mechanical strain produced by an applied electric field). However, these piezoelectric ceramics require high processing temperatures if thin films with dipole orientation are required. In addition, to gain the highest performance, lead-containing materials, such as lead zirconium titanate (PZT) [191,192], should be used. To counter these drawbacks, polymer piezoelectric materials have been proposed as substitutes. Polymer piezoelectric materials have significant advantages; in addition to their soft elasticity, both the materials and processing equipment required are inexpensive [193–195]. As a result, piezoelectric polymers enable the fabrication of flexible sensors [196–206], energy generators [207–210] and organic-based field-effect transistors [211] for the next generation of smart technology. Furthermore, although piezoelectric polymers have lower piezoelectric strain constants, they present better piezoelectric voltage constants (*g*, the electric field produced by a mechanical stress) due to the low dielectric permittivity of the polymer, as described in the following equation: *g* = *d*/(*ɛ*_o_*ɛ*_r_); where *g* is piezoelectric voltage constant, *d* is piezoelectric strain constant, *ɛ*_o_ is the vacuum permittivity, *ɛ*_r_ and is the relative permittivity of the piezoelectric materials. This indicates that piezoelectric polymers are particularly well suited to sensor applications involving the detection of pressure or human motion [212,213]. Here, we are focusing on the mechanical sensing aspect; however, some piezoelectric polymers can be used in other sensory applications, such as pyrometers, flame and thermal sensors. These sensors will be discussed in a later part of the section.

**Figure 10.**
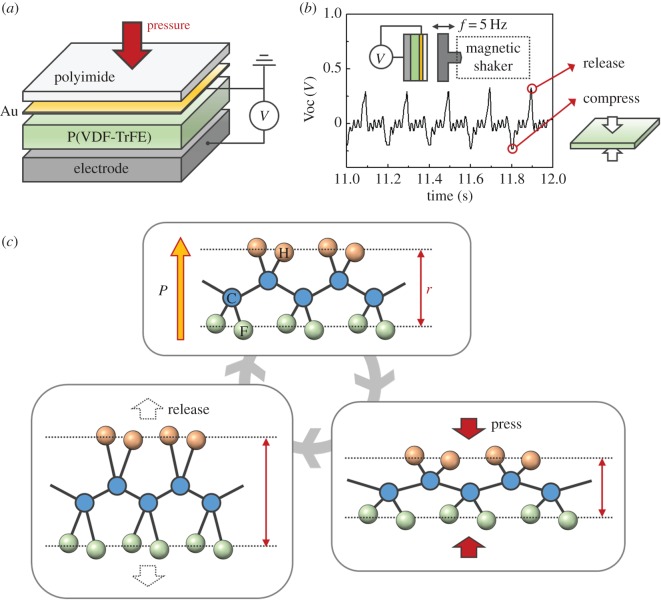
(*a*) Schematic of a P(VDF-TrFE)-based tactile sensor. (*b*) Output voltage of P(VDF-TrFE)-based tactile sensor when impacted with low-frequency (*f* = 5 Hz) vibrations using a magnetic shaker. Red circles indicate the deformation states of the piezoelectric polymer film. (*c*) Schematic drawing of the piezoelectric polymer-based tactile sensor mechanism based on atoms. Structure of PVDF in the all-trans configuration and its dipole moment (*P*). Red arrows indicate the separation (r) between hydrogen (orange circle) and fluorine (green circle) atoms.

Figure 10*a* presents a schematic of a tactile sensor using the piezoelectric polymer polyvinylidene fluoride-trifluoroethylene (P(VDF-TrFE)). The design of a typical piezoelectric tactile sensor is that of a two-plate capacitor with the piezoelectric polymer as the dielectric material, where the applied force induces a charge across the capacitor which is sensed by a voltage or charge amplifier circuit. Figure 10*b* illustrates typical output voltage characteristics of the piezoelectric polymer tactile sensor under low-frequency vibration (5 Hz) [136]. This device shows an alternating current shaped output signal with a peak voltage 0.3 V under 0.1% s^–1^ of strain rate. The magnitude and frequency of applied stress should be considered significant since the shape of the output signal is determined by those factors [214].

The atomic structure of piezoelectric polymer materials is the most significant factor in understanding the sensing mechanism of piezoelectric polymer tactile sensors. Figure 10*c* shows the atomic structure and dipole moment (*P*) of polyvinylidene fluoride (PVDF), which is one of the most cited and widely used piezoelectric polymer materials [193,194] due to its high electromechanical coupling property, approximately 28 pC^−1^ N [215]. PVDF has a spatially symmetrical arrangement of hydrogen and fluorine atoms along the polymer chain. In this spatial arrangement, the difference between electron preference (electronegativity) of atoms generates polarization in the molecules [216]. P(VDF-TrFE), which is used as a sample tactile sensor (figure 10*a*), is a copolymer of PVDF, and the additional side group helps the polymer retain an all-trans conformation. Figure 10*c* depicts the sensing mechanism of the piezoelectric polymer-based tactile sensor. In the case of PVDF, polarization of molecules decreases as a result of applied pressure because the magnitude of polarization is proportional to the distance (*r*) between hydrogen and fluorine atoms. Positive voltage output occurs when the pressure is released, because the restoration of the film increases the distance between atoms. Other piezoelectric polymers containing atoms giving rise to different dipole arrangements also generate voltages in a similar way.

Although there are commercially available PVDF and P(VDF-TrFE) films for pressure sensors and energy harvesting, owing to their relatively high sensitivity [217] their use is limited to sensing dynamic forces and pressures only. In other words, piezoelectric polymer materials are inappropriate for static measurements. This is because the electrical charge generated in a piezoelectric material under static stress decays over time, depending on the dielectric constants, internal film resistance and conductivities of the connected materials involved [218–225]. Therefore, careful consideration regarding application specifications is necessary when choosing piezoelectric materials for sensors.

Tensile sensors for monitoring various kinds of motions have been researched. Razian *et al.* developed an in-shoe triaxial pressure transducer with a piezoelectric polymer, for the diagnosis of foot disorders [226]. P(VDF-TrFE)-based sensors can simultaneously measure the vertical and horizontal shear forces under the foot with average sensitivities of 20 pC N^–1^ and 2.2 pC N^–1^, respectively. Tanaka *et al.* [227] developed a haptic finger sensor to evaluate skin conditions using PVDF film. The variance of the signal and dispersion of power spectrum density in frequency domain was translated to give an index of skin roughness and hardness, respectively. As a result, relatively reliable agreement was observed between analysed data and clinical assessment from people with various skin disorders. Tanaka *et al.* [228] also developed a piezoelectric polymer-based palpation sensor for prostatic cancer and hypertrophy detection. To measure the stiffness of the prostate gland, a PVDF film-based sensor was inserted into the examinee's rectum. The results show that data obtained from the sensor were in good correlation with the doctor's conventional examination. Kim *et al.* [200] explored a piezoelectric polymer-based sensitized microgripper for micro-assembly and micro-manipulation. The microgripper, incorporating PVDF, presented reliable force feedback with high sensitivity and high signal-to-noise ratio at 1 Hz linear load with a resolution of 39.5 mN V^–1^. The PVDF-based microgripper sensor can be used to measure gripping forces of the order of micronewtons.

Piezoelectric polymer materials can be used not only for stress sensing, but also for other types of sensor applications, such as heat sensing, since some piezoelectric materials have pyroelectric properties. Pyroelectric materials have temperature-dependent dipole moments. As a result, these materials generate electrical charges on their surfaces in response to thermal energy [229,230]. Lee *et al.* developed a flexible piezoelectric and pyroelectric hybrid device using P(VDF-TrFE) [231]. This device contained a composite of polydimethyl-siloxane (PDMS) with carbon nanotubes (CNTs) as a stretchable electrode, and grapheme nanosheets as a temperature gradient generator. In use, the magnitude of the applied force and temperature gradient could be found simultaneously from the magnitude of the output voltage of the hybrid device.

In summary, piezoelectric polymer materials are interesting candidates for mechanical force and temperature gradient sensing. The induced strain from applied force or external heat can be measured by analysing a voltage or charge across the piezoelectric material. Piezoelectric polymers, especially PVDF and its copolymers, are widely employed in commercial applications due to their wide bandwidth, fast electromechanical response, high voltage sensitivity, low acoustic and mechanical impedance, high strength and high impact resistance. However, some limitations, such as charge dissipation, should be considered when choosing suitable materials for sensing applications.

## 4. Concluding remarks and future prospects

We have presented a brief overview of EAPs for sensing by focusing on the materials used and the relevant sensing mechanisms. The main sensing properties of various EAPs discussed are summarized in tables 1–3, which should help evaluate different variants as materials for sensory applications.

**Table 1.** Materials selection guide for ionic EAPs.

type of EAPs	typical stimuli sensed	typical sensing range	typical working frequency or response time	typical signal readout	notes	references
conducting polymers	force or displacement	up to few per cent strain	0.1–100 Hz	—1 MPa stress produces 20–60 µV and 2000–6000 C m^−3^ —1000 C m^−3^ for 1% strain —>100 mV change in conductance —a few microampere —0.180 mV (3% strain)	potential drift due to environment changes. CPs are used in both free-standing and trilayer configurations.	[30–32,36,51,56]
	gas molecules	<10 ppm	few seconds			
ionic polymer–metal composites	displacement (strain)	up to 10% in strain	microseconds to seconds or up to hundreds of hertz	approximately 100 mV (200 N load)	potential drift due to environment changes	[84,232]
carbon nanotubes	force	up to several hundred MPa	milliseconds	∼75 nA (200 MPa load)	generally displays sharp current peaks	[105,109]
	gas molecules	∼0.01 ppm	2–10 s	few microsecond (NH_3_ detection)	generally displays high sensitivity

**Table 2.** Materials selection guide for electronic EAPs.

type of EAPs	typical stimuli sensed	typical sensing range	typical working frequency/response time	typical signal readout	notes	references
dielectric elastomers	strain	strain: 300%	<50 Hz (potential higher sensing frequency than the current value)	0–300 nF	used mainly for sensing mechanical strain, commercially established	[134,147,233]
liquid-crystal elastomers	compression	compressive strain >0.6	∼10 Hz	10–40 mV	only a few studies have been reported so far	[172,180,181]
	bending	displacement of a few millimetre	0.3–9 Hz	∼50 nC m^−1^		
piezoelectric polymers	pressure	<150 MPa	0.001–10^9^ Hz	∼0.013 V N^−1^	signal attenuates when measuring static force	[234]
	heat	20–180°C	n.a.	∼ 8 V/°K	n.a.

**Table 3.** Summary comparing the pros and cons of the major EAPs discussed in this review.

	EAPs for sensing	
	pros	cons
ionic EAPs		
conducting polymers	chemical stability, miniaturization, facile fabrication process, low-cost, low-weight, biocompatibility, soft, multiformable (sheet, film, tubular, trilayer), response to mechanical, electrical, chemical and thermal stimulation	insufficient adhesion onto substrate, fragile upon mechanical and thermal loading
ionic polymer–metal composites	low-weight, biocompatibility, miniaturization, soft, large produced voltage signal, sensitive to large bending deformation, ability to work in wet environments	hysteresis, sensitive to moisture and temperature during operation, operation limited to low temperature due to liquid electrolyte, slow response
carbon nanotubes	high surface area to volume, high sensitivity, miniaturization, directional mechanical and electrical properties	expensive, slow response, difficult to control intrinsic properties during fabrication
electronic EAPs		
dielectric elastomers	large sensing range, low-cost, light-weight, stability in many working cycles, and capability as self-sensing actuators	defect-sensitive, rare for stress measurement, not sensitive to compression in normal direction, may be affected by temperature
liquid-crystal elastomers	potential for high strain changes, potential high sensitivity to strain	complex and expensive synthesis and processing of the materials
piezoelectric polymers	wide bandwidth, fast electromechanical response, relatively low-power requirements, high generative forces	relatively low output performance originating from charge dissipation. Some materials need to be stretched and poled to gain higher output

Similar to other advanced technologies, EAPs will face some challenges before wide-scale deployment becomes a possibility:

(i) EAPs have the potential to be manufactured in various forms ranging from fibres, to films, to fabrics and strips [1,10]. EAPs are also being developed in the field of MEM technology [14,15]. For sensory applications, the continued implantation of sensor devices based on EAPs can improve ease of manufacture, mechanical flexibility and quality of signal output. Nonetheless, sensors for practical use must reach operational parameters reliably. Standards for both research purposes and mass-production are yet to be reached: this is probably due to the diversity of EAPs. There are many factors to consider, such as geometries, chemical compositions of materials, fabrication procedures and electromechanical coupling properties.
(ii) Maintaining the sensor specificity with appropriate packaging methods and materials is a major issue (particularly for ionic EAPs, as many of them need to work in moist conditions) [10]. Potential approaches for packaging or sealing the EAPs to prevent or retard degeneration are still in development [1,235], and have yet to meet industrial requirements.
(iii) Methods for the conveyance of sensory information from sensors to other recipients has yet to be developed, particularly for biomedical applications [236,237]. Recent efforts [238–240] have been made to develop EAPs that are compatible with neuron interfaces. There is hope that the neuron system can be used for communicating sensory information from EAP-based sensors.

Nonetheless, EAPs are attractive materials that will play a significant role in sensor-related technologies in the near future. There can be no doubt that inspiring stories about research and commercialization related to EAP sensors will continue.
